# Thermosensitive Hydrogel for Controlled Delivery of PAD4 Inhibitor YJ-2 in Diabetic Wound Healing

**DOI:** 10.3390/pharmaceutics18010135

**Published:** 2026-01-22

**Authors:** Kai Wang, Ayijiang Taledaohan, Liujia Chan, Yu Lu, Yijiang Jia, Yuji Wang

**Affiliations:** 1 Department of Medicinal Chemistry, College of Pharmaceutical Sciences of Capital Medical University, Beijing 100069, China; 2 Beijing Area Major Laboratory of Peptide and Small Molecular Drugs, Engineering Research Center of Endogenous Prophylactic of Ministry of Education of China, Beijing Laboratory of Biomedical Materials, Laboratory for Clinical Medicine, Capital Medical University, Beijing 100069, China; 3 Department of Pharmacology, University of Maryland, Baltimore, MD 21201, USA

**Keywords:** PAD4 inhibitor, thermosensitive hydrogel, NETs, diabetic wound healing

## Abstract

**Background**: Diabetic wound healing is hampered by persistent inflammation and excessive neutrophil extracellular traps (NET) formation. Peptidylarginine deiminase 4 (PAD4) is a key enzyme driving this pathology. This study developed a thermosensitive chitosan/β-glycerophosphate hydrogel for the local delivery of a novel PAD4 inhibitor, YJ-2, to promote diabetic wound repair. **Methods**: A YJ-2-loaded hydrogel (CGY) was synthesized and characterized. *In vitro* studies used HaCaT cells and macrophages to assess proliferation, migration, NETs (via H3cit), and polarization. Efficacy was evaluated in diabetic C57 mouse wound models. **Results**: CGY exhibited temperature-sensitive gelation and sustained YJ-2 release. *In vitro*, YJ-2 inhibited NETs formation, reduced pro-inflammatory markers, promoted HaCaT migration, and induced M2 macrophage polarization. *In vivo*, CGY treatment significantly accelerated wound closure. **Conclusions**: Local hydrogel delivery of the PAD4 inhibitor YJ-2 effectively mitigates inflammation and NETs, promoting healing in diabetic wounds. This strategy represents a promising targeted therapy for diabetic wounds.

## 1. Introduction

Diabetes mellitus is a prevalent chronic metabolic disorder that affects hundreds of millions of individuals worldwide [1]. Among its complications, impaired wound healing represents one of the most challenging conditions, severely compromising patients’ quality of life and imposing a considerable burden on healthcare systems [2]. The delayed wound healing observed in diabetes is influenced by multiple factors, including hyperglycemia, vascular lesions, neuropathy, immune dysfunction, and chronic inflammation. Among these, persistent inflammation is considered a central contributor to impaired healing and is closely linked to abnormal activation of macrophage-associated immune signaling [3].

The wound healing process typically progresses through four sequential phases: hemostasis, inflammation, proliferation, and maturation. However, in diabetic patients, thickening of the basement membranes in capillaries and arterioles restricts blood flow, thereby disrupting the physiological healing cascade [4,5]. The sustained inflammatory response further delays the transition into the proliferative and remodeling phases, leading to chronic non-healing wounds that remain open and are highly susceptible to infection.

Within the immune system, peptidylarginine deiminase 4 (PAD4) is a calcium-dependent enzyme broadly expressed in neutrophils, monocytes, and macrophages [6,7]. PAD4 plays a pivotal role in immune defense by promoting the formation of neutrophil extracellular traps (NETs) [8]. NETs, composed of decondensed DNA and proteins, are designed to capture and eliminate pathogens. Nevertheless, in the diabetic microenvironment, excessive NET formation exacerbates chronic inflammation and is strongly associated with impaired wound repair [9,10,11]. Elevated PAD4 expression has been detected in neutrophils of diabetic patients, resulting in increased NETs accumulation in both circulation and wound tissues [12,13], which further intensifies local inflammation and delays tissue regeneration.

Significant progress has been made in the development of PAD4 inhibitors. Thompson et al. pioneered the synthesis of F-amidine and Cl-amidine, the first generation of potent, irreversible PAD4 inhibitors [14]. Previous PAD4 inhibitors such as Cl-amidine, TDFA, and 4B have shown potential in regulating NET formation and alleviating inflammation. However, these early-generation inhibitors possess notable limitations, including poor selectivity toward PAD4 over other PAD isoforms, rapid systemic clearance, and limited *in vivo* stability. To overcome these drawbacks, our group previously designed and synthesized a novel small-molecule PAD4 inhibitor, YJ-2, through rational structural modification. YJ-2 introduces a para-hydroxybenzamide backbone and an N-benzyl ornithine linker, which enhances both hydrophobic interactions within the PAD4 active pocket and hydrogen-bond stability. These structural optimizations markedly increase binding affinity and enzyme inhibitory efficiency. According to the currently reported data, the *in vitro* enzymatic inhibitory activity of YJ-2 against PAD4 is 0.836 ± 0.240 μM, which is superior to that of YW3-56 (2.86 ± 0.40 μM) or 4B (3.799 ± 2.058 μM). Therefore, in this study, we selected the novel synthetic compound YJ-2 as a candidate drug [15,16]. Although Wu et al. demonstrated that oral administration of Cl-amidine markedly reduced NET formation, alleviated inflammation, and accelerated wound healing in diabetic mice [17], Cl-amidine and its derivatives are prone to rapid metabolic degradation. To overcome this limitation, we employed a hydrogel-based drug delivery system. These findings provide a strong rationale for further exploration of PAD4 inhibitors as therapeutic agents for diabetes-associated chronic inflammation.

Given the central role of PAD4 in NET formation and inflammation, targeting PAD4 enzymatic activity represents a promising therapeutic strategy for diabetic wound management. By suppressing NETs generation, PAD4 inhibitors can attenuate persistent inflammation, mitigate tissue damage, and facilitate wound closure. Hydrogels, owing to their excellent biocompatibility, tunable mechanical properties, and drug-loading capacity, serve as ideal carriers for local delivery [18]. Importantly, hydrogels not only maintain a moist wound environment but also enable controlled drug release to sustain therapeutic concentrations at the wound site [19].

Recent studies have confirmed the therapeutic potential of hydrogel-mediated PAD4 inhibitor delivery. For example, Kaur et al. utilized alginate/methacrylamide/gelatin-based scaffolds to encapsulate the PAD4 inhibitor TDFA, demonstrating accelerated wound healing in diabetic mice [20]. However, early-generation inhibitors such as Cl-amidine and TDFA have pharmacokinetic limitations, while scaffold systems often require complex preparation. To address these challenges, we designed a thermosensitive chitosan/β-glycerophosphate (CS/β-GP) hydrogel. By combining this hydrogel platform with the novel PAD4 inhibitor YJ-2 developed by our group, we aimed to achieve superior therapeutic efficacy.

Given the critical role of PAD4 in sustaining chronic inflammation during diabetic wound healing, targeted inhibition of PAD4 via hydrogel-based local delivery represents a promising therapeutic strategy. Such an approach has the potential to improve wound healing outcomes while minimizing systemic exposure. In this context, the present study aimed to develop a thermosensitive chitosan/β-glycerophosphate hydrogel for the controlled local delivery of a novel PAD4 inhibitor, YJ-2, and to systematically evaluate its therapeutic efficacy and underlying mechanisms in diabetic wound healing.

## 2. Materials and Methods

### 2.1. Materials

Boc-Orn (Cbz)-OH, tetrahydrofuran (THF), 1-hydroxybenzotriazole (HOBt), dicyclohexylcarbodiimide (DCC), benzylamine, N-methylmorpholine (NMM), p-hydroxybenzoic acid, Pd/C, 2-chloroacetimidate hydrochloride, N,N-diisopropylethylamine (DIPEA), chitosan (CS), and β-glycerophosphate (β-GP) were purchased from Aladdin Biochemical Technology Co., Ltd. (Shanghai, China) Rabbit anti-PAD4 antibody (AB96758), rabbit anti-H3cit antibody (AB281584), rat anti-LY6G antibody (AB210204) were purchased from Abcam (Waltham, MA, USA). Rabbit anti-ACTIN antibody (GB15003), rabbit anti-Ki67 antibody (GB111141), HRP-conjugated goat anti-rabbit IgG (GB23303), CY3-conjugated goat anti-rabbit IgG (GB21303), FITC-conjugated goat anti-rat IgG (GB22302) were purchased from Wuhan Seville Biotechnology Co., Ltd. (Wuhan, China).

### 2.2. Synthesis of Yj-2

**Figure 1:**
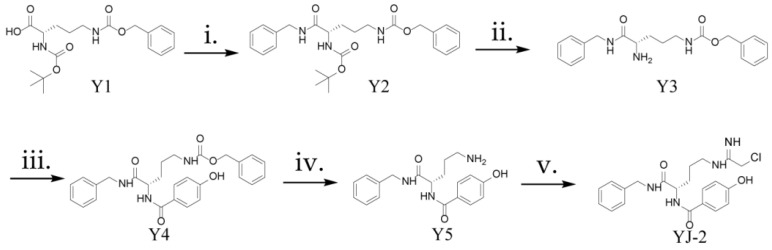
Synthetic route of YJ-2: i. benzylamine, DCC, HoBt; ii. 4N HCl/EA, ice bath; iii. p-hydroxybenzoic acid, DCC, HoBt; iv. H2, Pd/C; v. ethyl 2-chloroacetimidate, anhydrous methanol, DIPEA.

The synthetic route of YJ-2 is illustrated in Figure 1. Detailed procedures are provided in the Supplementary Materials.

### 2.3. Characterization of Yj-2

YJ-2 was characterized using multiple techniques. 1H NMR spectra were recorded on a Bruker 300 MHz instrument (Bruker, Billerica, MA, USA) with DMSO-*d6* as solvent. Fourier-transform infrared (FTIR) spectra were obtained using lyophilized powder on a Nicolet iS5 spectrometer (Nicolet, Madison, WI, USA). Ultraviolet–visible (UV–Vis) absorption measurements were performed using a UV-2600 spectrophotometer (Shimadzu, Kyoto, Japan). The absorbance of YJ-2 was recorded at a characteristic wavelength of 254 nm. Calibration curves were established using standard YJ-2 solutions with known concentrations. All measurements were conducted at room temperature.

### 2.4. Preparation of CGY

Chitosan (2% *w*/*v*) was dissolved in 0.1 M HCl. β-GP (50% *w*/*v*) was dissolved in deionized water. Under ice-bath conditions, β-GP solution was added dropwise into the CS solution with continuous stirring until homogeneity was achieved. For drug-loaded hydrogels, lyophilized YJ-2 was dispersed in the mixture (final loading up to 20 mg/mL).

### 2.5. Hydrogel Characterization

#### 2.5.1. FTIR Analysis

Lyophilized samples of CG, YJ-2, and CGY were analyzed by FTIR.

#### 2.5.2. Scanning Electron Microscopy (Sem)

Both hydrogels were placed on a silicon wafer and freeze-dried. The dried hydrogels were immersed in liquid nitrogen for 1 min and fractured with forceps. The samples were then sputter-coated with a 20 nm gold–palladium layer using a JEOL JFC-1600 Auto Fine Coater (JEOL, Tokyo, Japan) at 20 kV, 30 mA, and 200 mTorr argon pressure for 60 s. SEM imaging was performed with a HITACHI S-4800 (Hitachi, Tokyo, Japan) at 20 kV, clearly revealing the porous hydrogel architecture as well as the incorporated YJ-2 particles.

#### 2.5.3. Rheological Characterization

The rheological properties of the hydrogels were evaluated using a rotational rheometer (MCR302, Anton-Paar (Graz, Austria), PP25 geometry). Prepared hydrogel samples were placed between parallel plates, and time sweep measurements were performed at 25 °C under fixed strain and frequency, with a shear rate ranging from 0.1 to 150 s^−1^. The storage modulus (G′) and loss modulus (G″) were recorded as a function of frequency to characterize the viscoelastic behavior of the hydrogels.

#### 2.5.4. *In Vitro* Release of CGY

YJ-2 exhibited a characteristic absorption peak at 254 nm. For the release study, 2 mL of CG was placed in a 15 mL EP tube, followed by incorporation of 10 mg YJ-2 with thorough mixing. The mixture was then incubated in a 37 °C water bath to induce gelation. Subsequently, 10 mL of PBS was carefully added around the hydrogel at room temperature. At designated time points, 1 mL of supernatant was collected daily and replaced with an equal volume of fresh PBS [21]. The absorbance at 254 nm was measured using a microplate reader, and YJ-2 concentrations were calculated based on a standard curve to generate the cumulative release profile.

### 2.6. In Vitro Assays

#### 2.6.1. Cell Culture

HaCaT keratinocytes were obtained from American Type Culture Collection (ATCC) (Manassas, VA, USA). HaCaT keratinocytes were cultured in DMEM supplemented with 10% FBS in a humidified incubator at 37 °C with 5% CO_2_ (HERACELL VIOS 160i, Thermo Fisher). All cell handling procedures were performed under sterile conditions in a biosafety cabinet (1300 SERIES A2, Thermo Fisher, Waltham, MA, USA).

#### 2.6.2. MTT Assay

Cell proliferation in response to hydrogels was assessed using the MTT [3-(4,5-dimethylthiazol-2-yl)-2,5-diphenyltetrazolium bromide] assay.

One milliliter of CG was transferred into a 15 mL centrifuge tube and incubated in a 37 °C water bath to allow gelation. The formed hydrogel was gently detached from the tube wall using a spatula, followed by the addition of 10 mL of PBS for immersion for 24 h. The resulting extract was then filtered through a 0.22 μm membrane to obtain the hydrogel extract for subsequent experiments.

HaCaT cells were digested with trypsin, centrifuged, and resuspended in complete medium at a density of 4 × 10^4^ cells/mL. Cells were seeded into 96-well plates at 100 μL per well and incubated for 12 h in a CO_2_ incubator. Subsequently, 25 μL of YJ-2 at various concentrations, hydrogel extracts, or their combination was added to each well and incubated for 24 h. After treatment, 25 μL of MTT solution (5 mg/mL) was added per well and incubated for 4 h. The supernatant was then removed, and 150 μL of DMSO was added to each well. Plates were shaken at room temperature for 15 min, and absorbance was measured at 490 nm and 570 nm using a microplate reader.

#### 2.6.3. Scratch Assay

Cell migration was evaluated using a scratch assay. HaCaT cells were digested with trypsin, centrifuged, and resuspended in complete medium at a density of 5 × 10^5^ cells/mL. Cells were seeded into 6-well plates at 2 mL per well and incubated for 24 h in a CO_2_ incubator until a confluent monolayer was formed. A sterile 200 μL pipette tip was used to create a linear scratch perpendicular to the well surface. Detached cells were removed by washing with PBS, and images were captured to serve as the 0 h baseline. Cells were then cultured in DMEM containing 2% FBS, supplemented with 500 μL of YJ-2, hydrogel extracts, or their combination. Images were taken at 12 h and 24 h, and the wound area was quantified using ImageJ2 software.

#### 2.6.4. Anti-Inflammatory Assay

The anti-inflammatory activity of YJ-2 was evaluated using RAW 264.7 macrophages. Cells were seeded in 12-well plates at a density of 5 × 10^5^ cells/mL (1 mL per well) and incubated overnight. After medium replacement, cells were divided into Blank, Control, YJ-2 (15, 30, 60 mg/mL), and LPS groups. Appropriate amounts of YJ-2 were added to the designated wells to reach the target concentrations, while 20 μL of LPS (0.5 mg/mL) was added to all groups except Blank and Control, followed by 24 h incubation.

Supernatants were collected for ELISA to measure the levels of IL-6, IFN-γ, and TNF-α. In addition, cells from the Control, YJ-2 (30 mg/mL), and LPS groups were harvested and analyzed by flow cytometry to determine the proportion of CD206^+^ macrophages, a marker of M2 polarization.

#### 2.6.5. Anti-Net Assay

The anti-NET activity of YJ-2 was evaluated using murine neutrophils. Culture plates were pre-coated overnight with poly-L-lysine. ICR mice were anesthetized and sacrificed by cervical dislocation, and femurs were harvested. Bone marrow was flushed out with PBS, filtered, and centrifuged to isolate cells. Neutrophils were then purified using a commercial kit and seeded at 5 × 10^5^ cells per well. Cells were randomly divided into five groups: Control, PMA, YJ-2, hydrogel extract, and YJ-2 + hydrogel extract. YJ-2 and/or hydrogel extract were added to the designated groups, while equal volumes of PBS were added to the others. After 2 h of incubation, NET formation was induced by adding PMA to all groups except the control group, followed by an additional 2 h incubation.

Cells were collected by centrifugation (500× *g*, 5 min) and washed with PBS. NETs were fixed with 4% paraformaldehyde for 15 min, followed by PBST and PBS washes. After blocking with 5% BSA for 30 min at room temperature, samples were incubated with primary antibodies at 4 °C overnight. Following washes, cells were incubated with fluorescent secondary antibodies for 2 h at room temperature in the dark. DAPI was used for nuclear counterstaining. Samples were mounted with antifade reagent and imaged using a confocal laser scanning microscope.

### 2.7. In Vivo Studies

#### 2.7.1. Diabetic Mouse Model

Male C57BL/6 mice (7 weeks old) were purchased from Beijing Vital River Laboratory Animal Technology Co., Ltd. (Beijing, China). The animal study in this work was approved by the Institutional Animal Care and Use Committee of Capital Medical University, and the ethics number is AEEI-2025-957. Humane care of animals was given in the animal studies and followed the protocol and the Regulations on Laboratory Animal Welfare issued by Chinese Ministry of Science and Technology.

Male C57BL/6 mice were randomly divided into five groups and acclimated for one week under standard conditions with free access to food and water. Diabetes was induced by intraperitoneal injection of streptozotocin (STZ, 50 mg/kg, dissolved in citrate buffer, pH 4.5) once daily for five consecutive days under light-protected conditions. Fasting blood glucose was measured after treatment, and mice with glucose levels exceeding 280 mg/dL were considered successfully diabetic.

#### 2.7.2. Wound Healing Model

Mice were anesthetized with 2% isoflurane, and dorsal hair was removed. Two full-thickness excisional wounds with an 8 mm diameter were created on the dorsal skin of each mouse using a biopsy punch. Treatments were applied topically to the wounds on days 0, 3, 5, 7, and 9 during the 11-day treatment period, with a fixed application volume of 0.1 mL per wound for all groups. Wound images were captured every two days to monitor the healing progression, and wound areas were quantified using ImageJ software. Wound areas were quantified using ImageJ software. On day 11, mice were euthanized, and wound tissues were harvested for histological analysis and protein extraction.

#### 2.7.3. Histological Staining

Paraffin-embedded tissue sections were deparaffinized and rehydrated through routine procedures, followed by H&E, Masson’s trichrome, Ki67 immunohistochemistry (IHC), and immunofluorescence staining.

For H&E staining, sections were sequentially stained with hematoxylin and eosin, dehydrated, cleared, and mounted with neutral resin.

For Masson’s trichrome staining, sections were pretreated overnight in solution A, followed by sequential staining, differentiation with designated reagents, dehydration, clearing, and mounting.

For Ki67 IHC, antigen retrieval was performed, and endogenous peroxidase activity was blocked. Sections were then blocked with serum, incubated with primary antibody overnight at 4 °C, followed by incubation with secondary antibody. DAB was used for chromogenic detection, and hematoxylin was used for counterstaining. After dehydration and clearing, sections were mounted and observed under a light microscope.

For immunofluorescence staining, antigen retrieval and serum blocking were followed by overnight incubation with primary antibody mixtures at 4 °C. Sections were then incubated with fluorescently labeled secondary antibodies and counterstained with DAPI. After quenching autofluorescence, sections were mounted with antifade reagent and imaged under a fluorescence microscope. The excitation/emission wavelengths were as follows: DAPI (330–380 nm/420 nm), FITC (488 nm/515–555 nm), Cy3 (510–560 nm/590 nm), and Cy5 (608–648 nm/672–712 nm).

#### 2.7.4. Western Blotting

Total protein was extracted from wound tissues using standard procedures. Briefly, tissues were washed with PBS, minced, and homogenized in lysis buffer, followed by incubation at 4 °C for 30 min. Lysates were centrifuged at 12,000 rpm for 10 min, and the supernatants were collected as protein samples. Protein concentrations were determined, mixed with reducing loading buffer, and denatured at 95 °C for 10 min. Equal amounts of protein were separated by SDS-PAGE and transferred onto ethanol-activated PVDF membranes under wet transfer conditions (constant current, 300 mA, 30 min).

Membranes were blocked with 5% non-fat milk at room temperature for 30 min and then incubated overnight at 4 °C with primary antibodies. After washing with TBST, membranes were incubated with the corresponding HRP-conjugated secondary antibodies (1:5000) for 30 min at room temperature. Following thorough washing, membranes were immersed in ECL detection reagent for 1 min, and signals were captured using a chemiluminescence imaging system. Original images were saved in TIFF format.

#### 2.7.5. Statistical Analysis

All data were processed using GraphPad Prism 9.5.0 software. Statistical differences among groups were evaluated by one-way ANOVA, and a *p*-value of less than 0.05 was regarded as statistically significant. Results are presented as mean ± standard deviation (SD).

## 3. Results

### 3.1. Synthesis and Characterization of Yj-2

The PAD4 inhibitor YJ-2 was synthesized as outlined in Figure 1. Briefly, under standard coupling conditions (EDC·HCl, HOBt, DMF, NMM), precursor Y1 was condensed with benzylamine, followed by Boc deprotection (4 M HCl/EA) to yield Y3. Y3 was subsequently coupled with p-hydroxybenzoic acid to generate intermediate Y4, which was subjected to hydrogenolysis (Palladium on carbon (Pd/C), H2/MeOH) to obtain Y5. Finally, the free amine of Y5 reacted with 2-chloroethanimidamide hydrochloride (DIPEA, MeOH) to afford the final compound YJ-2.

The structure of YJ-2 was confirmed using multiple analytical techniques, details are provided in the Supplementary Materials.

Mass spectrometry (ESI-MS): *m*/*z* = 417.3 [M+H]^+^.

^1^H NMR (300 MHz, DMSO-*d6*): *δ* (ppm) = 10.12 (s, 1H), 10.03 (s, 1H), 9.53 (s, 1H), 9.17 (s, 1H), 7.82 (d, *J* = 8.7 Hz, 2H), 7.26 (m, *J* = 7.2, 6.5 Hz, 5H), 6.81 (d, *J* = 8.7 Hz, 2H), 4.41 (m, 3H), 4.29 (d, *J* = 6.0 Hz, 2H), 3.28 (d, *J* = 6.3 Hz, 2H), 1.88–1.56 (m, 4H).

UV spectrum: A characteristic absorption peak was observed at 254 nm, consistent with the predicted structure of YJ-2.

FTIR (KBr): *ν* (cm^−1^) = 3320 (br, N-H, Amide/Amidine), 3024 (w, C-H, Aromatic), 2930, 2860 (C-H, Aliphatic), 1686 (s, C=O, Amide I), 1606 (m, N-H bend/C-N stretch, Amide II coupled with Aromatic C=C), 1510 (C=C, Aromatic ring), 1250 (C-N), 731 (C-Cl).

These data collectively confirmed the successful synthesis and structural integrity of YJ-2.

### 3.2. Preparation and Characterization of Thermosensitive Hydrogel

**Figure 2:**
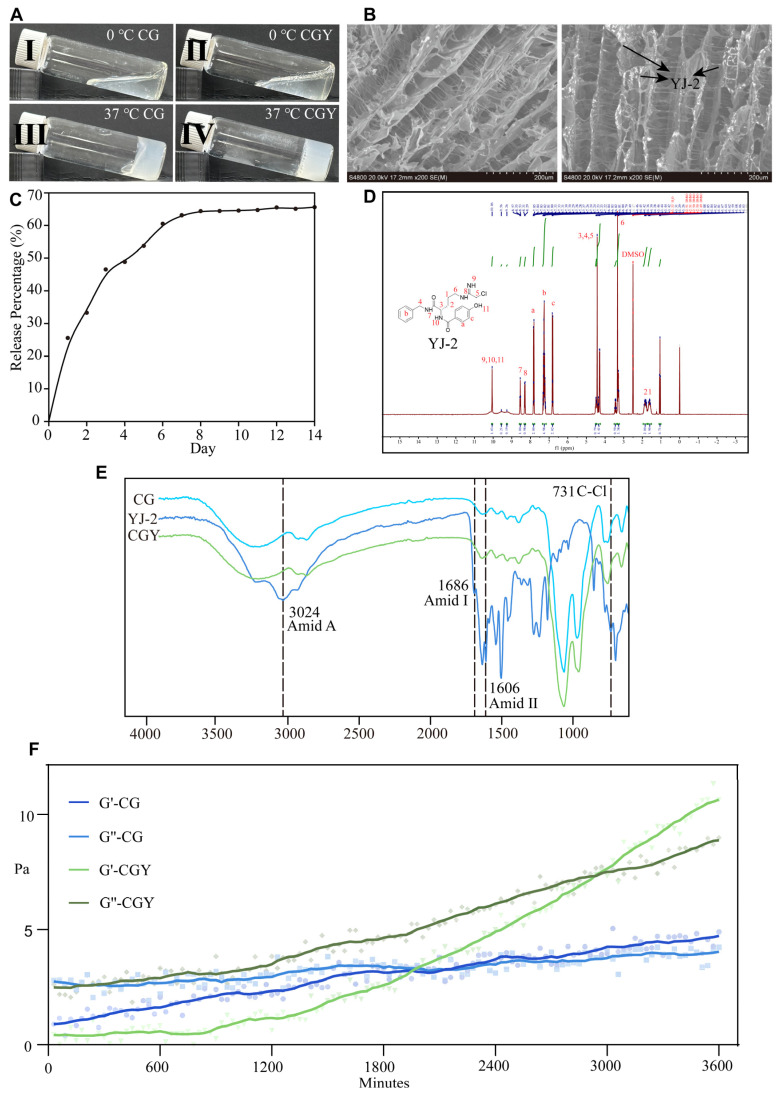
Characterization of CG and CGY. (**A**) I—0 °C CG. II—0 °C CGY. III—37 °C CG. IV—37 °C CGY. (**B**) SEM images of CG (**left**) and CGY (**right**). (**C**) Drug release profile of CGY. (**D**) 1H NMR spectrum of YJ-2 (300 MHz, solvent: DMSO-*d6*). (**E**) FTIR spectrum of CG, YJ-2 and CGY. (**F**) Time sweep of CG and CGY at 37 °C (G′: storage modulus, G″: loss modulus).

A thermosensitive chitosan/β-glycerophosphate (CS/β-GP) hydrogel was fabricated and evaluated as the delivery system for YJ-2. The blank hydrogel is referred to as CG, and the YJ-2-loaded hydrogel is referred to as CGY. The concentrations of chitosan and β-GP were selected based on previously reported thermosensitive CS/β-GP hydrogel systems [22], while the YJ-2 loading concentration was determined by considering its PAD4 inhibitory activity, cytocompatibility, and suitability for local wound application. This formulation remains liquid at ambient temperature for convenient application and undergoes rapid gelation at physiological temperature, ensuring excellent biocompatibility and sustained release (Figure 2A).

Morphology. Scanning electron microscopy revealed a regular porous architecture of CG. Incorporation of YJ-2 did not significantly alter the microstructure, SEM analysis revealed particle-like features within the hydrogel matrix, which may arise from lyophilization-induced phase separation or network reorganization (Figure 2B).

Drug release profile. The release kinetics of YJ-2 from CGY were determined by UV absorbance at 254 nm. The *in vitro* drug release profile demonstrated that YJ-2 was released from the CS/β-GP hydrogel in a biphasic manner—characterized by an initial burst release of ~ 45% of the load YJ-2 within the first 72 h followed by a sustained release up to 14 days. This release pattern is highly consistent with the pharmacodynamic requirements of the wound healing process. The early burst phase provides sufficient local drug concentration to suppress the acute inflammatory response and inhibit excessive NET formation, which typically peaks within the first three days after injury. The subsequent sustained-release phase maintains a moderate YJ-2 level during the tissue proliferation and remodeling stages (days 4–14) (Figure 2C). Such temporal drug release behavior prolonged anti-inflammatory and pro-regenerative effects are crucial for restoring normal healing dynamics. Therefore, the release of YJ-2 from the hydrogel maximizes its biological efficacy throughout the wound healing cascade. During the release experiments, no obvious macroscopic disintegration of the hydrogel was observed in PBS within 14 days.

FTIR analysis. Fourier-transform infrared spectroscopy demonstrated characteristic spectral changes upon YJ-2 loading. Specifically, the absorption band at 966.16 cm^−1^ shifted to 951.70 cm^−1^, likely due to hydrogen bonding interactions between YJ-2 and the hydrogel. Additionally, an increased peak intensity was observed between 1633.41–1257.36 cm^−1^, along with the emergence of a new absorption band at 1603.52 cm^−1^, attributed to the aromatic skeletal vibrations of YJ-2 (Figure 2D). Nevertheless, the overall spectrum of CGY remained similar to that of CG, indicating minimal disruption to the polymeric network—consistent with the SEM observations.

Rheological properties. Rheological testing further confirmed the thermosensitive gelation behavior. At 37 °C, the storage modulus (G′) surpassed the loss modulus (G″) at 37.5 min for CG, whereas CGY exhibited gelation at 51.5 min. These findings demonstrate that YJ-2 incorporation slightly delays gelation but does not compromise the thermoresponsive properties of the hydrogel (Figure 2E).

Together, these results establish that CG provides a suitable platform for the encapsulation and controlled release of YJ-2.

### 3.3. Cell Experiments

#### 3.3.1. Cell Viability

**Figure 3:**
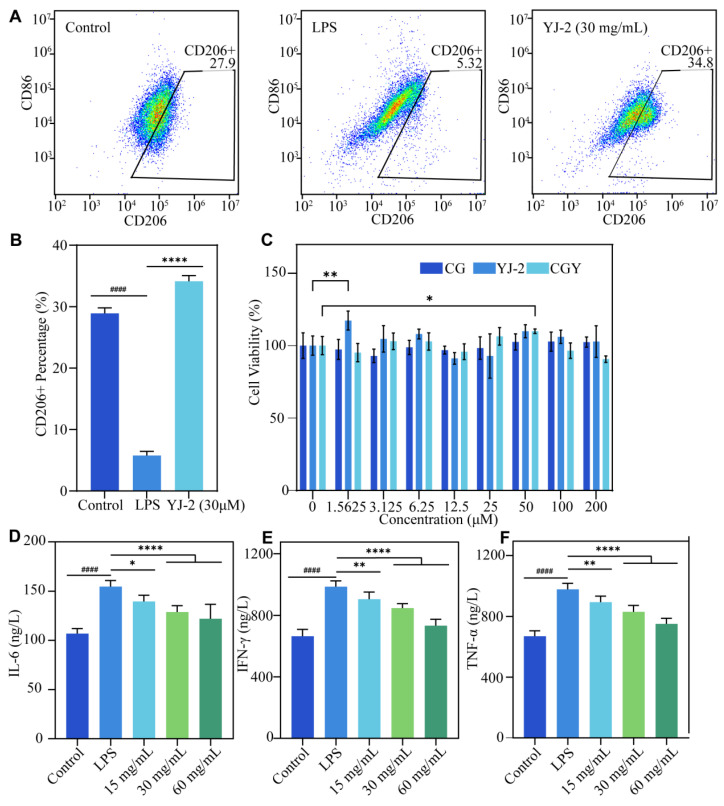
YJ-2 promotes cell proliferation, induces M2 macrophage polarization, and suppresses inflammatory cytokines. (**A**) Macrophage polarization assay. (**B**) Percentage of CD206^+^ cells (*n* = 3). Compared with the LPS group: ****, *p* < 0.0001. Compared with the Control group: ####, *p* < 0.0001. (**C**) HaCaT cell proliferation assay (*n* = 6). Compared with the control group: *, *p* < 0.05; **, *p* < 0.01. (**D**) IL-6 concentration. (**E**) IFN-γ concentration. (**F**) TNF-α concentration (*n* = 6). Compared with the LPS group: *, *p*< 0.05; **, *p* < 0.01; ****, *p* < 0.0001. Compared with the Control group: ####, *p* < 0.0001.

MTT assays demonstrated that YJ-2 promoted HaCaT keratinocyte viability at appropriate concentrations, without exerting cytotoxic effects even at higher concentrations (Figure 3C). This suggests that YJ-2 possesses favorable biocompatibility.

#### 3.3.2. Cell Migration

**Figure 4:**
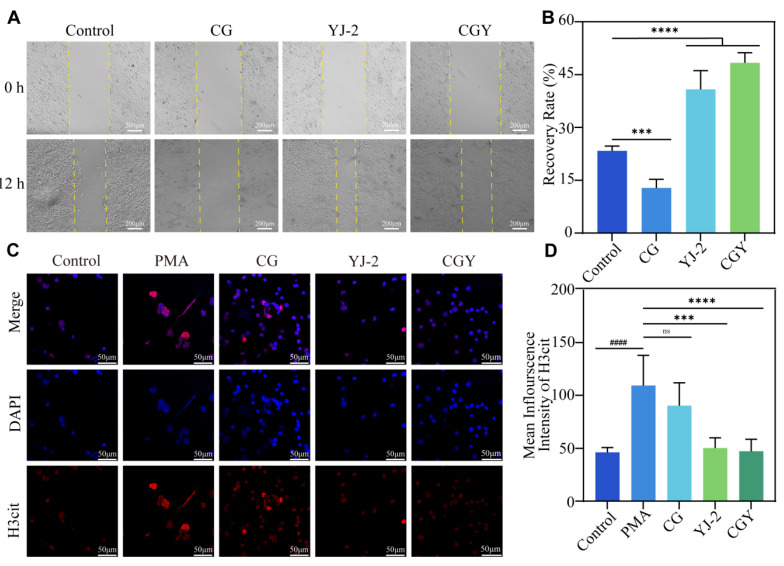
YJ-2 accelerates scratch wound closure and inhibits NET formation. (**A**) Scratch assay of HaCaT cells (YJ-2: 40 μM, 5×). (**B**) Scratch closure rate at 12 h (*n* = 5). Compared with the control group: ***, *p* < 0.001; ****, *p* < 0.0001. (**C**) *In vitro* anti-NET assay of mouse neutrophils. (**D**) Mean fluorescence intensity of H3cit protein in mouse neutrophils (*n* = 5). Compared with the PMA group: ns, *p* > 0.05; ***, *p* < 0.001; ****, *p* < 0.0001. Compared with the Control group: ####, *p* < 0.0001.

HaCaT cells play a crucial role in wound healing; therefore, a scratch assay was performed on confluent HaCaT monolayers to evaluate the effects of CG, YJ-2 and CGY on cell migration, thereby assessing their potential impact on wound repair (Figure 4A). Both free YJ-2 and CGY extracts significantly enhanced HaCaT cell migration. Specifically, treatment with YJ-2 increased the scratch closure rate from 23.35% to an average of 44.57%, representing a 90.85% improvement (Figure 4B). These findings suggest that YJ-2 possesses the potential to promote wound healing.

#### 3.3.3. Anti-Inflammatory Activity

Cytokine analysis revealed that LPS markedly induced inflammatory factor production in RAW 264.7 cells, whereas YJ-2 significantly inhibited this effect. CD206 is widely recognized as a specific surface marker of M2 macrophages, whose expression is closely associated with anti-inflammatory activity, tissue repair, and immune regulation. Flow cytometry results showed that 28.9% of untreated RAW 264.7 cells differentiated into M2 macrophages. This proportion decreased sharply to 5.76% following LPS stimulation, but co-incubation with YJ-2 restored the M2 fraction to 34.1% (Figure 3A,B). Additionally, YJ-2 demonstrated significant inhibitory effects on the inflammatory cytokines IFN-γ, IL-6, and TNF-α compared with the LPS group, with maximal inhibition rates of 25.65%, 21.12%, and 23.32%, respectively. These results strongly indicate that YJ-2 effectively counteracted the inhibitory effect of LPS on M2 polarization, confirming its potent anti-inflammatory activity and supporting its role in wound repair (Figure 3D–F).

#### 3.3.4. Inhibition of NET Formation

Aberrant NET formation is a critical factor contributing to impaired diabetic wound healing. Citrullinated histone H3 (H3cit), generated by PAD4, serves as a key inducer of NETs. Thus, the PAD4 inhibitor YJ-2 may promote wound healing by suppressing H3cit and thereby reducing NET formation. To verify the anti-NET activity of YJ-2, immunofluorescence staining of H3cit was performed. In confocal images (Figure 4C), H3cit (red) co-localized with chromatin (blue) in all groups. In the PMA-induced and hydrogel-only groups, pronounced chromatin decondensation, neutrophil rupture, and elevated H3cit fluorescence intensity were observed compared with the control. In contrast, both YJ-2–treated groups showed significantly reduced fluorescence intensity and minimal chromatin decondensation (Figure 4D). Quantitative analysis demonstrated that YJ-2 reduced H3cit expression in murine neutrophils by 55.40%, confirming its ability to inhibit NET formation.

Collectively, these cellular assays demonstrate that YJ-2 enhances keratinocyte proliferation and migration, alleviates inflammatory responses, and inhibits NET formation—thereby establishing a strong mechanistic foundation for its role in promoting diabetic wound healing.

### 3.4. Animal Experiments

**Figure 5:**
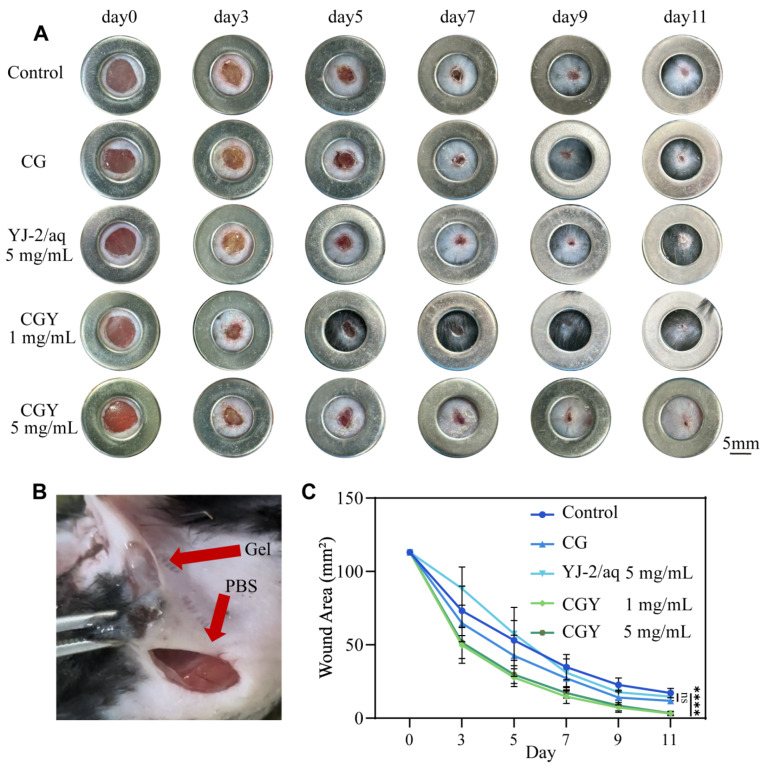
CGY forms a membrane at the wound site and promotes wound healing. (**A**) Wound healing progression in diabetic mice treated with different formulations over 11 days. (**B**) Wounds treated with hydrogel and PBS. (**C**) Quantitative analysis of wound area (*n* = 16). Compared with the control group: ns, *p* > 0.05; ****, *p* < 0.0001.

After confirming that YJ-2 promoted cell migration and inhibited NET formation *in vitro*, we further investigated the effect of CGY on wound healing in diabetic mice. Figure 5B shows the wound condition after treatment with CG and PBS. Compared with the control group, a distinct gel membrane can be clearly observed at the wound site in the hydrogel-treated group. Representative wound images over 11 days are shown in Figure 5A. From day 3, wounds treated with CGY exhibited markedly enhanced closure, and by day 11, wounds were nearly completely healed. And as shown in Figure 5C, quantitative analysis of wound areas indicated that the wound healing rates on day 11 were 81.41% and 79.73% in the groups with YJ-2 loading concentrations of 1 mg/mL and 5 mg/mL, respectively, compared with the control group.

**Figure 6:**
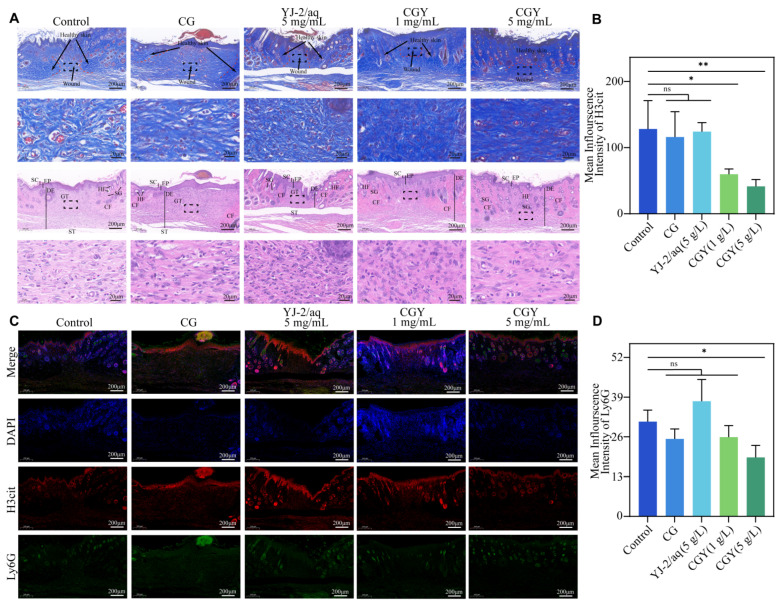
Histological and immunofluorescence staining of mouse wound sections. (**A**) Masson’s trichrome and H&E staining of wound tissue sections from mice, GT = granulation tissue, SC = stratum corneum, EP = epidermis, DE = dermis, ST = Subcutaneous tissue, SG = sebaceous glands, CF = collagen fibers, HF = hair follicles. (**B**) Mean fluorescence intensity of H3cit in wound tissues (*n* = 3). Compared with the control group: ns, *p* > 0.05; *, *p* < 0.05; **, *p* < 0.01. (**C**) Immunofluorescence images of H3cit and Ly6G in wound sections. (**D**) Mean fluorescence intensity of Ly6G in wound tissues (*n* = 3). Compared with the control group: ns, *p* > 0.05; *, *p* < 0.05.

Histological examination of wound tissues collected on day 11 was performed to evaluate the therapeutic effects of CGY. H&E and Masson’s trichrome staining (Figure 6A) revealed that wounds treated with CGY exhibited more compact and well-organized collagen fibers compared with the control group, in which collagen appeared loosely distributed and irregular. The presence of compact collagen bundles in the hydrogel-treated group suggests accelerated tissue maturation and improved structural integrity of the regenerated skin. In addition, increased sebaceous gland formation was observed in the CGY-treated wounds, indicating enhanced skin appendage regeneration.

**Figure 7:**
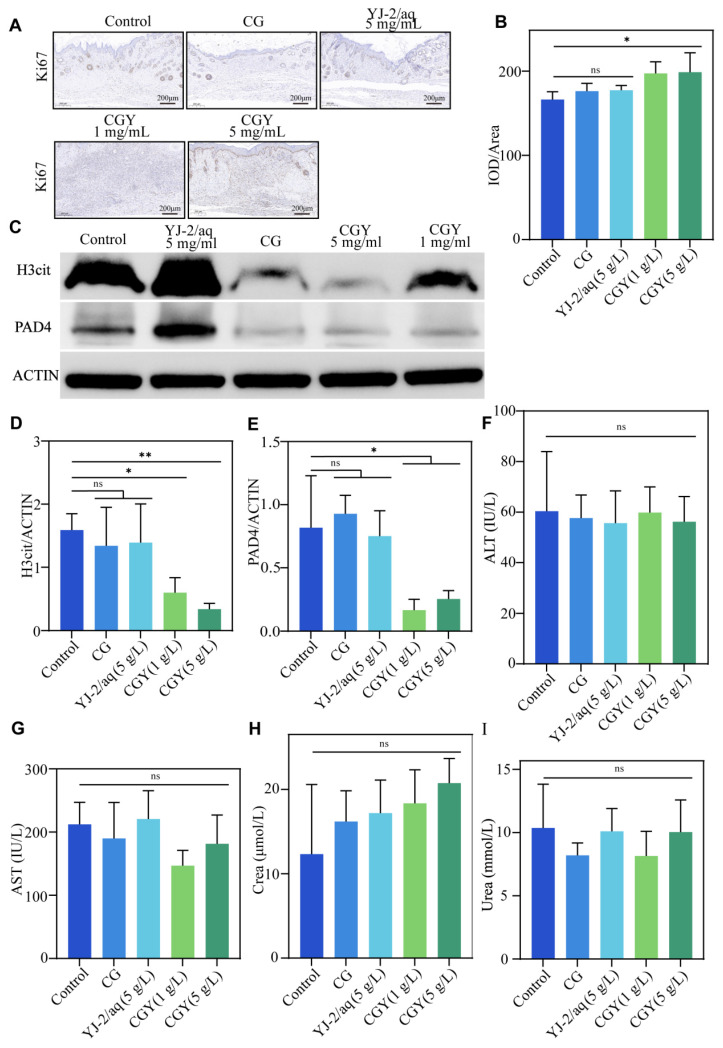
Immunohistochemistry, Western blot analysis, and routine blood tests of mouse wounds. (**A**) Immunohistochemical staining of Ki67 in wound sections. (**B**) Mean optical density of Ki67 in wound sections. (**C**) Western blot analysis of wound tissues from mice. (**D**) Relative gray value analysis of H3cit in wound tissues. (**E**) Relative gray value analysis of PAD4 in wound tissues (*n* = 3). Compared with the control group: ns, *p* > 0.05; *, *p* < 0.05; **, *p* < 0.01. (**F**–**I**) Serum biochemical parameters in mice: ALT, AST, Crea, and Urea (*n* = 4). Compared with the control group: ns, *p* > 0.05.

Immunohistochemical staining for Ki67, a proliferation marker, demonstrated higher protein expression in hydrogel-treated wounds. Quantitative analysis (Figure 7A,B) showed that CGY at 1 mg/mL and 5 mg/mL increased Ki67 expression by 18.68% and 19.58%, respectively, suggesting enhanced cellular proliferative activity that favors re-epithelialization and wound healing.

Immunofluorescence staining for H3cit and Ly6G (NETs-associated markers) further confirmed the inhibitory effect of CGY (Figure 6C). As shown in Figure 6B,D, CGY reduced H3cit expression by 53.28% and 67.67% and Ly6G expression by 16.36% and 37.82% at 1 mg/mL and 5 mg/mL YJ-2 loading, respectively. In contrast, wounds treated with YJ-2 aqueous solution displayed an upward trend in H3cit and Ly6G protein expression.

Western blot analysis further confirmed these observations (Figure 7C–E). Treatment with CGY (1 mg/mL and 5 mg/mL) suppressed H3cit expression by 62.22% and 78.65% and PAD4 expression by 45.04% and 24.24%, respectively. These findings provide additional evidence of the potent anti-NET activity of YJ-2 when delivered via hydrogel.

**Figure 8:**
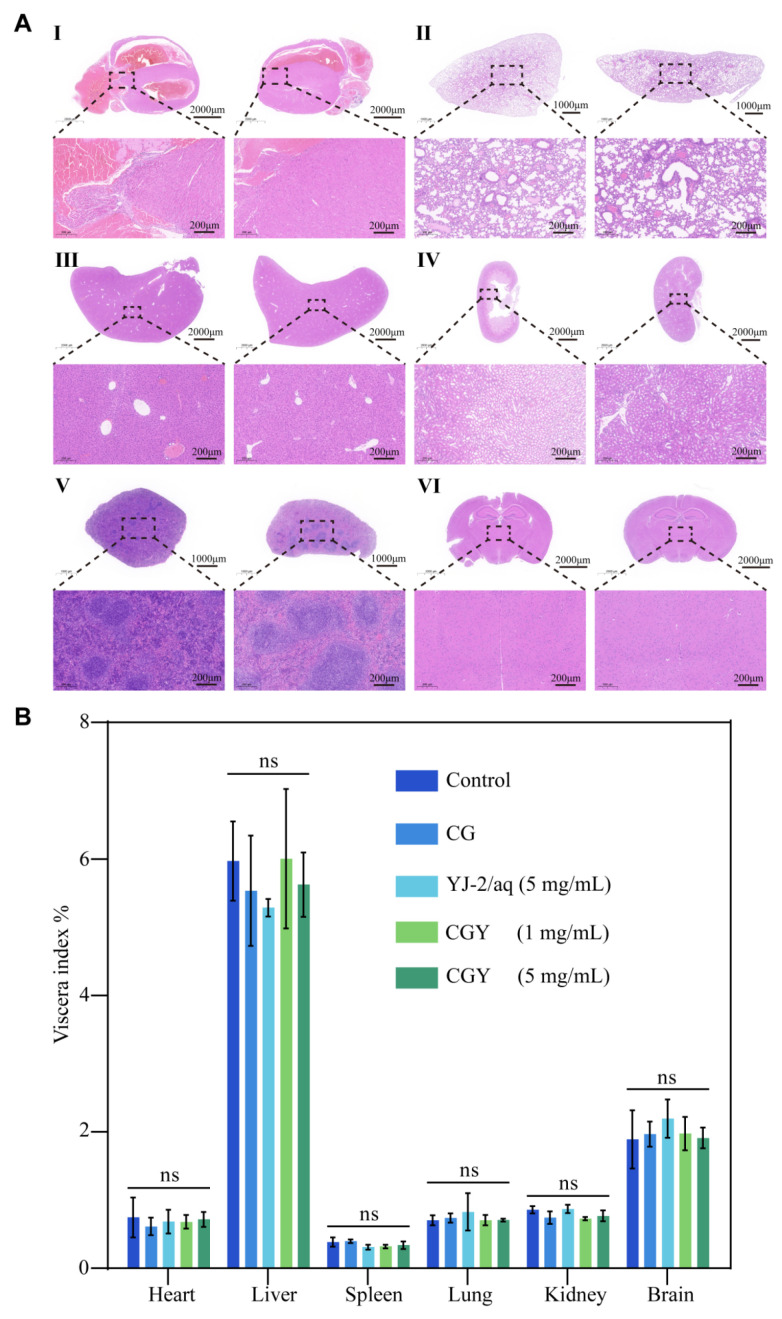
Biosafety evaluation. (**A**) H&E staining of major organs from mice in the control group (left) and 5 mg/mL treatment group (right). (I) Heart; (II) Lung; (III) Liver; (IV) Kidney; (V) Spleen; (VI) Brain. (**B**) Organ-to-body weight ratios of mice in each group (*n* = 4). Compared with the control group: ns, *p* > 0.05.

To evaluate systemic safety, histological sections of major organs (heart, liver, spleen, lung, kidney, and brain) were examined by H&E staining. No pathological abnormalities were observed in any group (Figure 8A). Blood biochemical analyses revealed no significant differences in serum ALT, AST, Crea, or Urea levels among groups (Figure 7F–I), and organ-to-body weight ratios also showed no significant changes (Figure 8B). These results collectively indicate that CGY possesses excellent biocompatibility and systemic safety.

Taken together, these *in vivo* findings demonstrate that CGY significantly accelerates diabetic wound healing, enhances collagen remodeling, promotes cell proliferation, and effectively suppresses NET formation without inducing systemic toxicity.

## 4. Discussion

Mechanistic studies demonstrated that hydrogel-mediated delivery of YJ-2 effectively inhibited PAD4 activity, reduced H3cit expression, and suppressed NET formation, thereby alleviating excessive inflammation and promoting HaCaT keratinocyte proliferation and migration. These results highlight the importance of sustained local PAD4 inhibition in improving the chronic wound microenvironment.

In contrast, treatment with YJ-2 aqueous solution resulted in increased H3cit and Ly6G expression, which may be attributed to rapid drug diffusion and a compensatory upregulation of PAD4 following transient inhibition [23]. The hydrogel formulation, by providing sustained and localized release of YJ-2 at lower concentrations, maintained effective PAD4 suppression without inducing excessive feedback regulation, leading to significantly improved wound healing.

Notably, the chitosan hydrogel alone also moderately promoted wound repair, consistent with its inherent biocompatibility and wound-supportive properties. In addition, diabetic wounds exhibit a dynamic pH microenvironment [19], and previous studies have shown that similar hydrogels display sustained release at pH 5.4 and 7.4, with accelerated release under acidic conditions. This pH-responsive behavior suggests that the hydrogel system may adapt to inflammatory wound states, further enhancing its therapeutic efficacy [24].

It is worth noting that in clinical wound repair, in addition to wound healing itself, scar formation is also an important challenge. Abnormal repair often leads to keloids or hypertrophic scars, the pathological mechanisms of which involve excessive proliferation of fibroblasts, activation of myofibroblasts, abnormal extracellular matrix (ECM) deposition, and persistent inflammatory response [25,26,27,28,29]. It has been proven in other previous studies that NETs and PAD4 are not only involved in delayed wound healing but also closely related to pathological scar formation. Excessive NETs and inflammatory factors can activate fibroblasts and stimulate profibrotic signaling pathways such as TGF-β1, leading to Col I expression, thereby inducing collagen deposition and scar formation [30,31,32,33,34,35]. Neutrophils have been reported to promote YAP transcription and expression, whereas a previously reported PAD4 inhibitor, YW-356, was shown to suppress YAP expression by more than 65%. Inhibition of PAD4 activity and NET formation may therefore attenuate YAP signaling, which is closely associated with fibroblast activation and fibrotic responses. Importantly, although transient activation of YAP contributes to normal wound repair, sustained or excessive YAP activation has been implicated in abnormal cell proliferation, excessive extracellular matrix deposition, and pathological fibrosis, ultimately leading to hypertrophic scar formation [36,37]. In parallel, anti-inflammatory M2 macrophages play a critical role in regulating tissue repair and determining the extent of scar formation, and modulation of macrophage polarization has been proposed as a promising clinical strategy to optimize wound healing and reduce hypertrophic scar (HTS) development. In this study, *in vitro* experiments demonstrated that YJ-2 markedly increased the proportion of M2 macrophages, which may contribute to limiting excessive collagen deposition [38,39]. Therefore, PAD4 inhibition achieved through sustained delivery may accelerate wound healing while reducing the occurrence of hypertrophic scars.

In addition, the hydrogel system itself may also have anti-scar potential. Biocompatible hydrogels can provide a moist healing environment, regulate mechanical tension at the wound site, and can be further designed for controlled release of anti-fibrotic factors. Especially chitosan-based hydrogels, due to their inherent anti-inflammatory and ECM regulatory properties, are considered to have advantages in inhibiting scar formation [40,41,42,43]. Therefore, CGY constructed in this study not only provides a new approach for diabetic wound repair but may also have potential value in scar prevention, which is worthy of further exploration in long-term animal models and clinical studies.

## 5. Conclusions

In this study, we developed a thermosensitive chitosan/β-glycerophosphate (CS/β-GP) hydrogel for the sustained delivery of the novel PAD4 inhibitor YJ-2. The hydrogel exhibited favorable physicochemical properties, excellent biocompatibility, and efficient drug release behavior. *In vitro*, YJ-2 promoted HaCaT cell proliferation and migration, suppressed NET formation, and induced M2 macrophage polarization. In diabetic mice, YJ-2-loaded hydrogels significantly accelerated wound closure, enhanced collagen remodeling, reduced inflammatory cell infiltration, and downregulated PAD4/H3cit expression, thereby improving wound repair.

Collectively, these findings demonstrate that hydrogel-mediated delivery of YJ-2 offers a promising therapeutic strategy for chronic diabetic wounds. Beyond enhancing wound healing, this approach may also reduce pathological scar formation, highlighting its potential translational value in regenerative medicine.

## Data Availability

The original contributions presented in this study are included in the article/Supplementary Materials. Further inquiries can be directed to the corresponding authors.

## Supplementary Materials

The following supporting information can be downloaded at https://www.mdpi.com/article/10.3390/pharmaceutics18010135/s1, Figure S1: The chemical structure of compound YJ-2; Figure S2: The MS spectra of synthetic intermediates Y2 and Y3.
