# Supplementary material for: Thermosensitive Hydrogel for Controlled Delivery of PAD4 Inhibitor YJ-2 in Diabetic Wound Healing
Source: Pharmaceutics. 2026 Jan 22;18(1):135. doi: 10.3390/pharmaceutics18010135 (PMC12845247; doi:10.3390/pharmaceutics18010135)
Supplement: Supplementary file 1 [file pharmaceutics-18-00135-s001.zip › pharmaceutics-4030897-supplementary.pdf]

Supporting Information

Thermosensitive Hydrogel for Controlled Delivery of PAD4 Inhibitor YJ-2 in Diabetic Wound Healing

Kai Wang [1,2], Ayijiang Taledaohan [3], Liujia Chan [1,2], Yu Lu [1,2], Yijiang Jia [1,2,*] and Yuji Wang [1,2,*]

1 Department of Medicinal Chemistry, College of Pharmaceutical Sciences of Capital Medical University, Beijing 100069, China; ito1g2@163.com (K.W.); clj970416@126.com (L.C.); luyu@mail.ccmu.edu.cn (Y.L.)
2 Beijing Area Major Laboratory of Peptide and Small Molecular Drugs, Engineering Research Center of Endogenous Prophylactic of Ministry of Education of China, Beijing Laboratory of Biomedical Materials, Laboratory for Clinical Medicine, Capital Medical University, Beijing 100069, China
3 Department of Pharmacology, University of Maryland, Baltimore, MD 21201, USA; tayijiang@som.umaryland.edu
* Correspondence: jiayijiang@ccmu.edu.cn (Y.J.); wangyuji@ccmu.edu.cn (Y.W.)

Synthesis of PAD4 Inhibitor YJ-2

1. Synthesis of Boc-Orn(Cbz)-NBzl (Y2)
Boc-Orn(Cbz)-OH (10 mmol) was dissolved in anhydrous THF (20 mL), followed by the addition of HOBt (12 mmol) under ice-bath conditions until complete dissolution. DCC (12 mmol) was added dropwise, and the mixture was stirred for 30 min to obtain reaction solution A. Benzylamine (12 mmol) was dissolved in anhydrous THF (20 mL) and added to solution A under ice-bath conditions, followed by the addition of N-methylmorpholine (NMM, 1 mL) to adjust the pH to 8–9. The mixture was stirred in an ice bath for 1 h and then at room temperature for 8 h. TLC (CH_2Cl_2:MeOH = 20:1, v/v) confirmed the disappearance of Boc-Orn(Cbz)-OH. The precipitated DCU was removed by filtration, and the solvent was evaporated under reduced pressure. The residue was dissolved in ethyl acetate (EA, 50 mL) and sequentially washed three times with saturated $NaHCO_3$, NaCl, $KHSO_4$, NaCl, $NaHCO_3$, and NaCl solutions. The organic phase was dried over anhydrous Na_2SO4, filtered, and concentrated under reduced pressure to yield Boc-Orn(Cbz)-NBzl.

2. Synthesis of HCl·H-Orn(Cbz)-NBzl (Y3)
Boc-Orn(Cbz)-NBzl (10 mmol) was dissolved in a small amount of anhydrous EA, and 4 mol/L HCl/EA solution was added under ice-bath stirring. TLC (EA:H_2O:HAc = 4:1:0.1, v/v/v) confirmed complete consumption of the starting material. The reaction mixture was evaporated under vacuum, re-dissolved in EA, and concentrated again under vacuum, repeated three times to obtain HCl·H-Orn(Cbz)-NBzl.

3. Synthesis of p-Hydroxybenzoic acid-Orn(Cbz)-NBzl (Y4)
p-Hydroxybenzoic acid (10 mmol) was dissolved in anhydrous THF (20 mL), followed by the addition of HOBt (12 mmol) under ice-bath conditions until complete dissolution. DCC (12 mmol) was added dropwise, and the mixture was stirred for 30 min to obtain reaction solution A. HCl·H-Orn(Cbz)-NBzl (12 mmol) was dissolved in anhydrous THF (20 mL) and added to solution A, followed by NMM (1 mL) to adjust the pH to 8–9. The reaction was stirred for 1 h in an ice bath and then at room temperature for 8 h. TLC (CH_2Cl_2:MeOH = 20:1, v/v) confirmed complete consumption of *p*-hydroxybenzoic acid. The precipitated DCU was removed by filtration, and the solvent was evaporated. The residue was dissolved in EA (50 mL), sequentially washed as described above, dried over Na_2SO_2, filtered, and concentrated to yield *p*-hydroxybenzoic acid-Orn(Cbz)-NBzl.

4. Synthesis of *p*-Hydroxybenzoic acid-Orn-NBzl (Y5)
p-Hydroxybenzoic acid-Orn(Cbz)-NBzl (10 mmol) was dissolved in methanol, followed by the addition of Pd/C catalyst. The system was sealed, degassed, and hydrogenated under H_2 atmosphere at room temperature with stirring until TLC monitoring confirmed the disappearance of the starting material. The reaction mixture was filtered to remove Pd/C and concentrated under reduced pressure to yield *p*-hydroxybenzoic acid-Orn-NBzl.

5. Synthesis of PAD4 inhibitor YJ-2 (*p*-Hydroxybenzoic acid-Orn(Cl)-NBzl)

p-Hydroxybenzoic acid-Orn-NBzl (1 mmol) was dissolved in anhydrous methanol. Under ice-bath conditions, ethyl 2-chloroacetimidate hydrochloride (5 mmol) was added, and the pH was adjusted to 10 with DIPEA. The reaction was stirred at room temperature for 12 h. TLC (EA:H_2O:HAc = 4:1:0.1, v/v/v) confirmed complete consumption of the starting material. The solvent was evaporated, and the crude product was purified by C18 column chromatography to afford the PAD4 inhibitor YJ-2 (*p*-hydroxybenzoic acid-Orn(Cl)-NBzl).

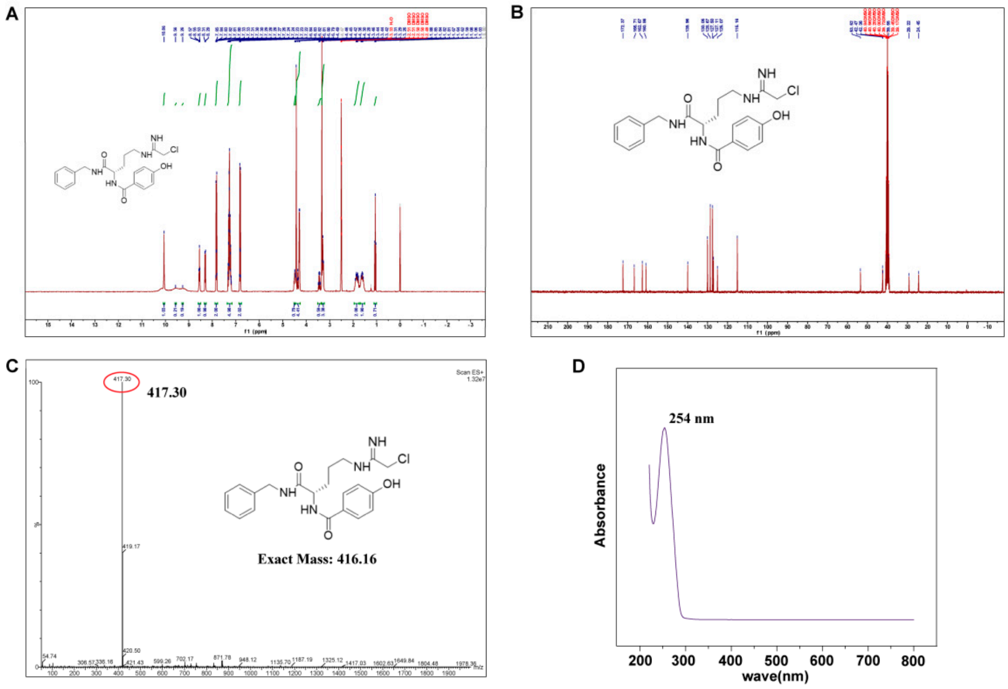

Figure S1. A. ^{1}H NMR spectrum of YJ-2 (300 MHz, solvent: DMSO-*d6*). B. ^{13}C NMR spectrum of YJ-2 (300 MHz, solvent: DMSO-*d6*). C. Mass spectrum of YJ-2 (ES^+). D. UV absorption spectrum of YJ-2.

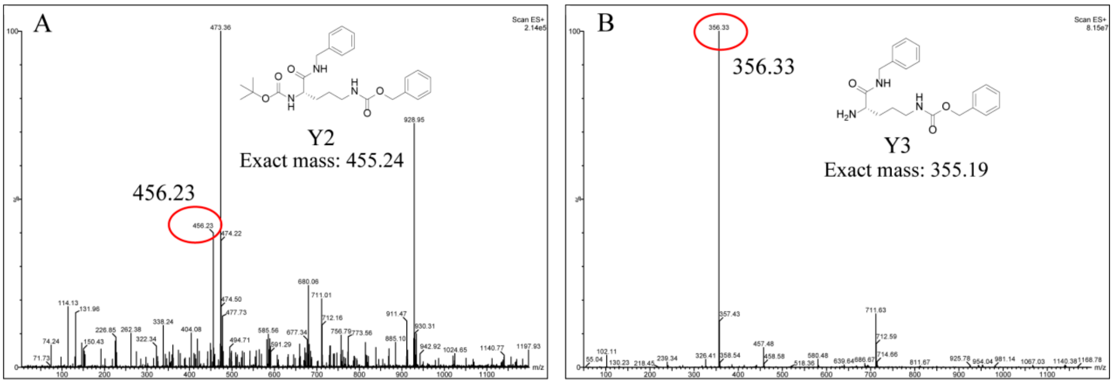

Figure S2. A. Mass spectrum of Y2 (ES^+). B. Mass spectrum of Y3 (ES^+).
